# *SAMHD1* is recurrently mutated in T-cell prolymphocytic leukemia

**DOI:** 10.1038/s41408-017-0036-5

**Published:** 2018-01-19

**Authors:** Patricia Johansson, Ludger Klein-Hitpass, Axel Choidas, Peter Habenberger, Bijan Mahboubi, Baek Kim, Anke Bergmann, René Scholtysik, Martina Brauser, Anna Lollies, Reiner Siebert, Thorsten Zenz, Ulrich Dührsen, Ralf Küppers, Jan Dürig

**Affiliations:** 1 Department of Hematology, University Hospital Essen, University of Duisburg-Essen, Essen, Germany; 2 Institute of Cell Biology (Cancer Research), University Hospital Essen, University of Duisburg-Essen, Essen, Germany; 3 Lead Discovery Center GmbH, Dortmund, Germany; 4 Center for Drug Discovery, Department of Pediatrics, Emory Center for AIDS Research, Emory University, Children’s Healthcare of Atlanta, Atlanta, GA USA; 5 Institute for Human Genetics, Christian-Albrechts-University Kiel and University Hospital Schleswig Holstein, Kiel, Germany; 6 Institute of Human Genetics, University of Ulm and University Hospital of Ulm, Ulm, Germany; 7 Department of Molecular Therapy in Haematology and Oncology, National Center for Tumor Diseases and German Cancer Research Center, Department of Medicine V, University Hospital Heidelberg, Heidelberg, Germany; 8 German Cancer Consortium (DKTK), Heidelberg, Germany

## Abstract

T-cell prolymphocytic leukemia (T-PLL) is an aggressive malignancy with a median survival of the patients of less than two years. Besides characteristic chromosomal translocations, frequent mutations affect the *ATM* gene, JAK/STAT pathway members, and epigenetic regulators. We here performed a targeted mutation analysis for 40 genes selected from a RNA sequencing of 10 T-PLL in a collection of 28 T-PLL, and an exome analysis of five further cases. Nonsynonymous mutations were identified in 30 of the 40 genes, 18 being recurrently mutated. We identified recurrently mutated genes previously unknown to be mutated in T-PLL, which are *SAMHD1*, *HERC1*, *HERC2*, *PRDM2*, *PARP10*, *PTPRC*, and *FOXP1*. SAMHD1 regulates cellular deoxynucleotide levels and acts as a potential tumor suppressor in other leukemias. We observed destructive mutations in 18% of cases as well as deletions in two further cases. Taken together, we identified additional genes involved in JAK/STAT signaling (*PTPRC*), epigenetic regulation (*PRDM2*), or DNA damage repair (*SAMHD1*, *PARP10, HERC1,* and *HERC2*) as being recurrently mutated in T-PLL. Thus, our study considerably extends the picture of pathways involved in molecular pathogenesis of T-PLL and identifies the tumor suppressor gene *SAMHD1* with ~20% of T-PLL affected by destructive lesions likely as major player in T-PLL pathogenesis.

## Introduction

T-cell prolymphocytic leukemia (T-PLL) is a rare leukemia with an aggressive disease course and a median survival of the patients of less than 2 years. Leukemic cells are characterized by expression of pan-T-cell markers with the unique feature of CD4 and CD8 co-expression in 25% of cases. A CD4^+^CD8^−^ phenotype is observed in 60% of patients, whereas a CD4^−^CD8^+^ phenotype is rare (~15%)^1,2^. Most T-PLL carry typical genetic alterations, namely inv(14)(q11q32), t(14;14)(q11;q32), or, less often, t(X;14)(q28;q11). These alterations, involving the TCRAD locus on chromosome 14q11, cause overexpression of the oncogenes *TCL1A* on chromosome 14q32 or *MTCP1* on chromosome Xq28^3–6^. Other frequent genetic lesions involve chromosome 8 (idic(8p), t(8;8)(p21;q11), trisomy 8q), and the *ATM* gene on chromosome 11 (11q2.23). *ATM* is deleted or mutated in up to 70% of cases^7–9^. Further recurrent deletions or losses occur on chromosomes 12p13 (*CDKN1B*), 6q, 17p13.1 (*TP53* locus), and 22q^10^. Sequencing analyses identified recurrent mutations in members of the JAK/STAT signaling pathway, as well as in epigenetic regulators^7,11–14^. Recent next-generation sequencing studies included whole-genome and exome sequencing^7^ as well as targeted deep sequencing^13,14^.

We characterized T-PLL by RNA sequencing, targeted capture sequencing, and whole-exome sequencing (WES) for somatic mutations, and by single-nucleotide polymorphism (SNP) arrays for detection of genomic imbalances in candidate regions. We identified recurrent mutations in *SAMHD1* in 6/33 cases (18%). Copy number losses were observed in two more patients. Other genes that exhibited recurrent mutations and/or copy number alterations were *HERC2*, *HERC1*, *PRDM2*, *PARP10*, *PTPRC*, and *FOXP1*.

## Materials and methods

### Patients and samples

Patient samples were obtained from archived material of the participating institutions. Patients were diagnosed between 2005 and 2012 in accordance with the WHO 2008 classification^2^. The study was approved by the ethical review committees of the Universities of Duisburg-Essen and Kiel (14-6080-BO and B295/11). All patients provided written informed consents according to the Declaration of Helsinki. The detection of an inv(14)/t(14;14) or t(X;14) by cytogenetic analysis and/or detection of *TCL1* or *MTCP1* breakpoints by FISH was required for inclusion into the study. Clinical data of 33 study patients are summarized in Table 1. Standard clinical criteria were applied for initiation of therapy^15^. RNA sequencing data and copy number analyses were assessed for 10 patients. As control, we sequenced T-cell RNA from five healthy donors. CD3^+^ T cells were enriched by magnetic cell separation (Miltenyi Biotech, Bergisch Gladbach). For 28 samples, including the 10 with RNA-sequencing analysis, DNA capture sequencing was performed. WES was carried out for five additional T-PLL. The assignment of samples and experiments is given in Supplementary Table 1.

**Table 1:** Clinical patient data

**Patient ID**	**Sex**	**Age at diagnosis (years)**	**Genetic group**	**T-PLL Immunophenotype**	**SAMHD1 status**	**Absolute WBC count at diagnosis (10** ^**9**^ **/L)**	**Absolute lymph. count at diagnosis (10** ^**9**^ **/L)**	**Other sites of involvement (except blood and BM)**	Type of Treatment*	**Allogenic transplant**	**CR at any time**	**Overall survival (months)**	**Death**	**Comorbidity**
**1**	F	74	inv(14)/t(14;14)	CD4+	Loss	719	62.7	None	1; 2; 3; 4	No	No	34	Yes	Prior T-PLL: Cervical carcinoma (radiotherapy, CR); immunoblastic lymphoma (CTX, CR)
**2**	F	64	t(X;14)	CD4+	Mutated	17.3	8.5	Skin	1; 2; 4	No	No	18	Yes	Rheumatoid arthritis; myociadial inf.; renal insuff, Std. III
**3**	M	62	inv(14)/t(14;14)	CD4+	Mutated	148	121	Spleen, liver, lymph nodes, skin	1; 2; 3; 4	No	No	7	Yes	None
**4**	M	69	inv(14)	CD4+	WT	n.a.	n.a.	n.a.	n.a.	No	No	0	Yes	n.a.
**5**	M	75	inv(14)	CD8+	WT	830	730	Spleen, liver, lymph nodes	1; 2; 3; 4	No	Yes	33	Yes	Ponsinfarction right; vasculare leukencephalopathie; hypertension; kidney-surgery right; polyneuropathy
**6**	F	41	t(X;?)	CD4+	WT	102	91	Spleen, lymph nodes, mediastinal	1; 2; 4	yes	no	12	Yes	None
**7**	F	51	t(14;14)	CD8+	WT	11.8	8	Spleen, liver, lymph nodes	1; 2; 3; 4	Yes	Yes	16	Yes	Diabetes mellitus; hypertension; adiposity; bronchial asthma
**8**	M	n.a.	t(X;14)		WT	n.a.	n.a.	n.a.	n.a.	n.a.	n.a.	33	Yes	n.a.
**10**	M	82	inv(14)	CD4+	WT	32	28	Spleen	1; 2; 4	No	No	5	Yes	Hypertension; renal insuff.; depression; prior to T-PLL: Prostate cancer
**11**	F	62	inv(14)	CD4+/CD8+	Loss	34	30	Spleen	1; 2; 3; 4	Yes	No	21	Yes	Hypertension; diabetes mellitus; ulcerative colitis
**12**	M	57	inv(14)	n.a.	WT	n.a.	n.a.	n.a.	n.a.	n.a.	n.a.	n.a.	n.a.	n.a.
**13**	F	53	inv(14)	n.a.	WT	n.a.	n.a.	n.a.	n.a.	n.a.	n.a.	n.a.	n.a.	n.a.
**15**	F	78	t(X;14)	CD4+	Mutated	n.a.	n.a.	n.a.	n.a.	No	n.a.	21	Yes	n.a.
**16**	M	47	inv(14)	n.a.	Mutated	n.a.	n.a.	n.a.	n.a.	n.a.	n.a.	n.a.	n.a.	n.a.
**17**	M	48	inv(14)	CD4+	WT	22	17.6	None	1; 2; 3; 4	No	No	20	n.a.	n.a.
**18**	M	n.a.	n.a.	n.a.	WT	n.a.	n.a.	n.a.	n.a.	n.a.	n.a.	n.a.	n.a.	n.a.
**19**	F	54	n.a.	n.a.	WT	181	n.a.	Lymph nodes	1; 2; 3; 4	Yes	Yes	158	No	None
**20**	F	55	n.a.	n.a.	WT	n.a.	n.a.	n.a.	n.a.	n.a.	n.a.	n.a.	n.a.	n.a.
**21**	M	n.a	n.a.	n.a.	WT	n.a.	n.a.	n.a.	n.a.	n.a.	n.a.	n.a.	n.a.	n.a.
**22**	F	69	inv(14)	n.a.	WT	n.a.	n.a.	n.a.	n.a.	n.a.	n.a.	n.a.	n.a.	n.a.
**24**	M	n.a	t(14;14)	n.a.	WT	n.a.	n.a.	n.a.	n.a.	n.a.	n.a.	n.a.	n.a.	n.a.
**25**	F	76	inv(14)	CD4+	WT	118	106	Spleen, lymph nodes	3	No	Yes	30	No	CLL
**26**	M	n.a.	inv(14)	n.a.	WT	n.a.	n.a.	n.a.	n.a.	n.a.	n.a.	n.a.	n.a.	n.a.
**28**	F	n.a.	inv(14)	n.a.	WT	n.a.	n.a.	n.a.	n.a.	n.a.	n.a.	n.a.	n.a.	n.a.
**29**	M	n.a.	inv(14)	n.a.	WT	n.a.	n.a.	n.a.	n.a.	n.a.	n.a.	n.a.	n.a.	n.a.
**30**	F	n.a.	t(X;14)	n.a.	WT	n.a.	n.a.	n.a.	n.a.	n.a.	n.a.	n.a.	n.a.	n.a.
**31**	F	n.a.	inv(14)	CD4+	Mutated	n.a.	n.a.	n.a.	n.a.	n.a.	n.a.	n.a.	n.a.	n.a.
**33**	F	64	inv(14)	CD4+	WT	30.2	28.4	Spleen, liver	3	No	No	17	No	Chronic HepC infection, liver cirrhosis; diabetes mellitus; renal insuff.; hypertension; heart insuff.; pulmonary hypertension; atrial fibrillation
**34**	M	71	n.a.	CD4+	WT	76	68	Skin	3	Yes	Yes	13	Yes	Hypertension
**35**	M	76	inv(14)	CD4+	WT	407	387	Spleen	3	No	No	19	No	Hypertension; hyperuricemia
**36**	M	70	t(X;14)	CD8+	UPD	185	180	Spleen, liver, lymph nodes,	3; 4	Yes	Yes	8	Yes	Hypertension; diabetes mellitus; renal insuff.
**37**	M	74	inv(14)	CD4+	Mutated	53	44	Lymph nodes, skin	4	No	No	51	No	M. Bechterew, psoriasis
**38**	F	74	inv(14)	CD4+	WT	79	72	Skin	1; 2; 3; 4	No	Yes	29	Yes	Hypertension

*WT* wildtype, *WBC* white blood cell, *Lymph.* lymphocyte, *BM* bone marrow, *CR* complete remission, *n.a.* not available.

*Type of treatment:

1 alkylators (chlorambucile, etc.),

2 purine analogs (Fludarabine, Pentostatin, etc.),

3 Anti-CD52 antibody (Alemtuzumab),

4 others

### Tumor cell enrichment

Details are given in the supplementary methods.

### RNA and DNA isolation

RNA and DNA were extracted from 1–2 × 10^7^ enriched tumor cells per sample. Details are given in the supplementary methods.

### Transcriptome sequencing

Sample libraries were prepared from RNA of isolated cells of 10 patients and five healthy blood donors. RNA sequencing (RNA-Seq) was performed on the HiSeq 2500 system with 2 × 101 bp paired-end reads (Illumina). Duplicate reads were removed and reads were quality filtered. Mutations were considered only if the particular position was covered at least 20-fold. For exclusion of polymorphisms, the dbSNP database was used. In general, single nucleotide variants were excluded if they matched i) a 1000 genomes entry and/or ii) exhibited an annotated variant allele frequency (VAF) above 1% and/or iii) occurred in one or more healthy donor samples. Filtering against healthy donor samples was performed to exclude sequencing artefacts. Database version dbSNP137 was used for data evaluation. Expression analysis of RNA-Seq data was performed with Partek Genomics Suite software, version 6.6; 2016 (Partek Inc., St. Louis, MO, USA)^16^. Further details are given in the supplementary methods. Data are available under GEO accession number GSE100882.

### Targeted capture sequencing

**Fig. 1:**
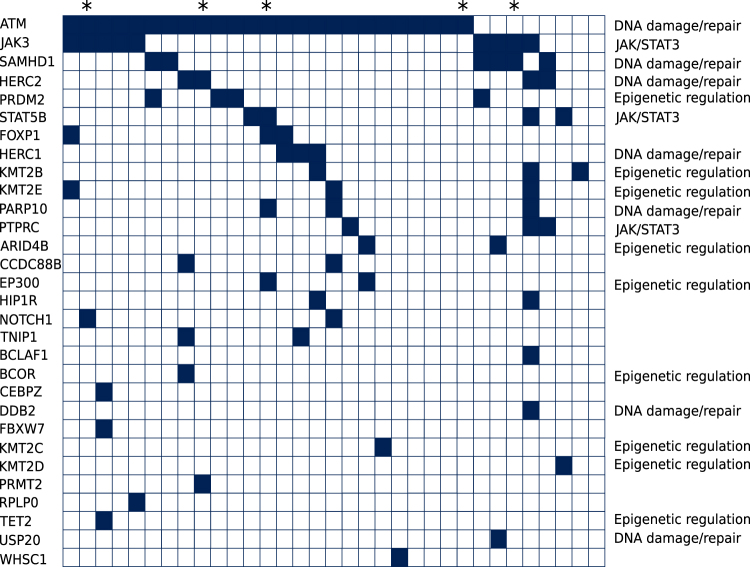
Distribution of mutated genes in the T-PLL cohort analyzed by targeted capture sequencing and WES Mutated genes are indicated as black fields for the 33 T-PLL that carried at least one mutated gene. * indicate the five cases for which results are generated by WES. Not listed are 10 genes without any mutations, which are *BCL11B*, *CBL*, *CUX1*, *ETV6*, *JAK1*, *MTOR*, *RUNX1*, *SUZ12*, *VOPP1*, and *ZRSR2*

To validate candidate mutations in genes identified by RNA-Seq, we selected 40 genes for which capture oligonucleotides for all coding exons of the respective genes were designed (Fig. 1). Further information is given in the supplementary methods. All variant calls originating from positions covered with less than 20 reads were removed. We excluded variants with less than 20% VAF and analyzed non-synonymous variants only. Polymorphisms were excluded as indicated above. Database version dbSNP137 was used for evaluation of capture sequencing data. Data are available under SRA accession number SRP111041.

### Whole-exome sequencing

For five T-PLL samples WES was performed. SNPs with entries in the 1000 genomes project^17^ were removed. We eliminated mutations if the respective position was covered with less than 20 reads. Only non-synonymous mutations were considered. We excluded variants with a VAF below 20%. Database version dbSNP147 was used for data evaluation. Data are available under SRA accession number SRP111041.

### Amplification and sequence analysis of mutations

To verify candidate mutations detected in *SAMHD1* by targeted capture sequencing or WES, we selected the respective mutated positions in this gene for three *SAMHD1*-mutated T-PLL. For all three cases, non-tumor DNA extracted from CD14^+^ and CD19^+^ cells was available. After PCR, amplicons were analyzed by Sanger sequencing (ABI3130 Genetic Analyzer; Applied Biosystems, Life Technologies). Primer sequences are available from the authors upon request.

### Copy number analysis

Copy number variation (CNV) analysis was carried out on Affymetrix SNP 6.0 microarrays (*n* = 10) and CytoScan HD arrays (*n* = 4; Affymetrix, Santa Clara, CA, USA). Further details are given in the supplementary information.

### Western blotting

For analysis of SAMHD1, equal amounts of protein lysates were separated by SDS-PAGE and transferred to a nitrocellulose membrane. Details are found in the supplementary information.

### Quantitative reverse transcription PCR

RNA was transcribed into cDNA with the high-capacity cDNA reverse transcription kit (Applied Biosystems). Details are given in the supplementary methods.

### Cell viability assay

For determination of the number of metabolically active cells, the CellTiter-Glo LuminescenT-cell Viability Assay (Promega, Fitchburg, WI, USA), which is based on quantification of ATP, was used. Details are described in the supplementary methods.

### Determination of cellular dNTP content

To obtain intracellular dNTPs, cell pellets prepared from 2 × 10^6^ cells of seven T-PLL samples comprising four *SAMHD1* mutated and three wild-type samples as well as three CD3^+^ MACS-enriched healthy donor samples were lysed. The extraction and quantitative measurement of intracellular dNTPs were conducted as reported previously^18^.

## Results

### Identification of mutations in T-PLL by RNA and DNA sequencing

#### Outline of the experimental design

For identification of recurrent mutations in T-PLL, we first performed RNA-Seq analysis of isolated tumor cells of 10 cases. We then selected 40 candidate genes from this analysis and studied them for somatic mutations in an extended cohort of 28 patients with a targeted DNA capture sequencing approach. Five additional T-PLL were analyzed by WES. For both DNA-sequencing analyses, we excluded mutations with low coverage (read counts < 20) at a respective position and occurring only at subclonal levels, as mutation detection is less reliable in these instances. The cutoff for subclonality was set to 20%. Polymorphisms were excluded (see Materials and Methods section).

#### Transcriptome sequencing

In the RNA-Seq analysis of 10 T-PLL, we obtained an average number of 9.5 million reads on the target region after quality filtering and duplicate removal. Focusing on variants with high sequence quality scores, we selected all genes carrying non-synonymous mutations and fulfilling the following criteria: (i) mutations in a gene in at least two T-PLL and (ii) a quality score of 1000, which is the highest possible value. The score is calculated as the −10log_10_ of the *p* value given by the AVADIS software. The 40 genes fulfilling the criteria (Fig. 1) were subjected to further analyses on the DNA level.

#### Capture sequencing

**Fig. 2:**
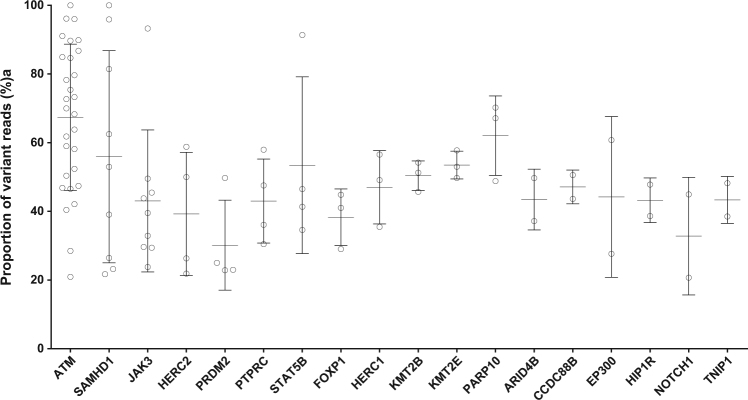
Variant allele frequencies of 18 mutated genes carrying at least two mutations Variant allele frequencies below 20% are not considered. Depicted are all mutations per gene. Bars indicate mean and S.D.

All coding exons of the 40 candidate genes selected from the RNA-Seq analysis were studied by a targeted deep sequencing approach in a total of 28 T-PLL, including the 10 samples on which RNA-Seq was performed. The average sequence coverage for the target region in the capture approach was 151, with 97% of target-region nucleotides covered at least 20 times. Twenty-nine of the forty genes (73%) showed non-synonymous mutations (Fig. 1). Most mutations appeared with VAF of 40–60%, indicating that they are clonal heterozygous mutations (Fig. 2; but note that SNVs with VAF below 20% were filtered out). Two genes (*ATM* and *SAMHD1*) showed several mutations with VAF between 90 and 100%, suggesting either homozygous or hemizygous clonal mutations. Ten of the forty genes selected from the RNA-Seq analysis were not found to be mutated in the targeted sequencing (and also not in a WES of five further cases, see below). The discrepancy to the RNA-Seq data is mainly due to the fact that we applied more stringent selection criteria in the DNA analysis (e.g., VAF ≥ 20%). Moreover, not all mutations identified by RNA-Seq were validated in the targeted sequencing approach, indicating some false-positive results in the RNA-Seq analysis, or that some alleles with subclonal mutations were preferentially transcribed.

#### WES

Five further T-PLL were analyzed by WES. On average, the target region of the exome was covered 75 times across all samples. Overall 88% of nucleotides were covered at least 20 times. After applying the above-mentioned filtering criteria, we first focused on the 40 genes selected for targeted sequencing. Ten of the forty genes (25%), namely *ATM*, *FOXP1*, *HERC1*, *HERC2*, *JAK3*, *NOTCH1*, *PARP10*, *PRMT2*, *SAMHD1*, and *STAT5B*, showed non-synonymous mutations (Fig. 1). Analyzing the complete WES data, we identified several previously unreported recurrently mutated genes (Supplementary Table 2). Three T-PLL displayed mutations in the ryanodine receptor 3 gene, *RYR3*, namely two replacement mutations and one splice site mutation. RYR3 can release calcium from the endoplasmic reticulum. *PARN* was mutated in two T-PLL. It encodes a poly(A)-specific ribonuclease, which degrades poly(A) tails of mRNAs. *PCLO*, coding for the piccolo presynaptic cytomatrix protein, was mutated in two T-PLL. This gene is also frequently mutated in diffuse large B-cell lymphomas^19,20^. One T-PLL also carried a mutation in the *IL2RG* gene, which is known to be recurrently mutated in T-PLL^7^.

Combining capture (*n* = 28) and WES data (*n* = 5), we observed in total mutations in 30 of the 40 genes (75%). *ATM* was the most frequently mutated gene, with 76% of cases harboring *ATM* mutations (Fig. 1), in line with prior studies^9^. Other genes known to be mutated in T-PLL^7,13,14^ were also recurrently mutated in our cohort, including *JAK3* (27% of cases mutated) and *STAT5B* (12%). Mutations in genes encoding epigenetic regulators, including four members of the KMT2 lysine-methyltransferase family, were observed in 3–9% of cases, and overall in 6 of the 33 cases. Mutations in other epigenetic regulators occurred at lower frequencies compared to previous reports, e.g., *BCOR* and *TET2* each in only 3% of cases (Fig. 1), whereas in prior studies, they were mutated in up to 9% and up to 17% of T-PLL, respectively^7,13,14^.

We also identified recurrently mutated genes known to be involved in tumorigenesis of various hematologic malignancies, but which have not been described in T-PLL yet. These are *SAMHD1*, *HERC1*, *HERC2*, *PRDM2*, *PARP10*, *PTPRC*, and *FOXP1*, which could be assigned to distinct functional categories (Fig. 1).

**Fig. 3:**
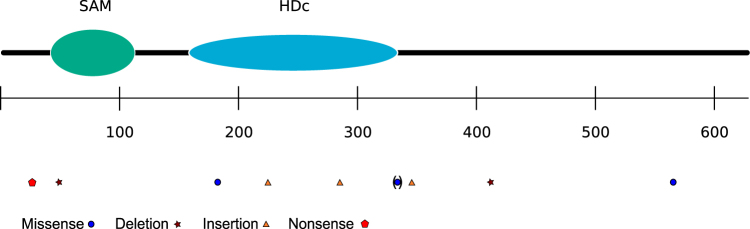
Pattern and distribution of mutations in SAMHD1 Depicted are all mutations. The missense mutation in brackets was also observed in hematopoietic non-tumor cells of a female patient carrying two *SAMHD1* mutations. The scale indicates the numbering of the amino acids. *SAM* sterile alpha motif, *HDc* Histidine (H)-Aspartate (D) containing

Besides *ATM*, the group of mutated genes related to DNA damage/repair included *SAMHD1* as the second most frequently mutated gene, which was mutated in 18% of cases. The pattern and distribution of these mutations are depicted in Figs. 1 and 3. *SAMHD1* encodes a dGTP-activated triphosphohydrolase, which regulates the cellular dNTP pool. Inactivating mutations in *SAMHD1*, which lead to increased dNTP levels, promote tumor cell survival. In our cohort, the majority of *SAMHD1* mutations are frameshift or nonsense mutations. Three of six mutated cases with four *SAMHD1* mutations could be used for verification studies by PCR and Sanger sequencing. All *SAMHD1* mutations observed in the next-generation sequencing approaches were confirmed. The pattern and distribution of these mutations are depicted in Fig. 3. An analysis of corresponding non-tumor DNA of these patients confirmed the somatic origin of three of the four mutations, whereas in the patient with two SNVs in the gene one of these was also detected in the non-tumor DNA, indicating that this is a germline variant. To test for mutual exclusivity of *SAMHD1* mutations with other recurrent mutations, we performed a contingency analysis, which revealed a significant negative association between *ATM* and *SAMHD1* mutations (Fisher’s exact test, *p* = 0.02).

The genes *HERC2* and *HERC1*, which are mutated in 12 and 9% of T-PLL, respectively, encode for E3 ubiquitin protein ligases. In both genes, we observed replacement mutations distributed along the whole length of the gene. *HERC2* and *HERC1* function as DNA repair genes^21^. *HERC1* is recurrently mutated in T-cell acute lymphoblastic leukemias (T-ALL)^22^.

*PARP10*, encoding the mono-ADP-ribosyltransferase PARP10, is mutated in 3/33 cases (9%). Two mutations lead most likely to loss of the protein or impaired protein function as they are a nonsense mutation and a missense mutation in the ubiquitin-interacting motif, which is part of the catalytic domain of the protein^23^. PARP10 is recruited to stalled replication forks and mediates cellular resistance to DNA damage, thereby promoting genomic stability^24^.

The most frequently mutated gene in the group of epigenetic regulators is *PRDM2*, with 12% of T-PLL harboring mutations. It is a member of a nuclear histone/protein methyltransferase superfamily and described as tumor suppressor gene, mutated in several types of cancers^25,26^. Microsatellite-instable tumors show frequent frameshift mutations of *PRDM2*^27^. Three of four mutations are predicted to change the amino-acid sequence in the coiled-coil domain of the protein. The lysine-methyltransferase genes *KMT2B* and *KMT2E* are mutated in three cases each (9%). All mutations are missense mutations, dispersed along the gene. KMT2B, mutated in several cancers^28^, is a methyltransferase, while KMT2E, which was initially grouped to the KMT2 family and is also mutated in various cancers^29^, seems to lack intrinsic histone methyltransferase activity.

*PTPRC*, encoding CD45, is expressed on all human hematopoietic cells, and has, among other functions, a role in inhibiting JAK/STAT signaling. A downregulation of CD45 is reported in T-ALL carrying *PTPRC* mutations^30^. We observed replacement mutations in *PTPRC* in 9% of cases. Two of three mutations were located in the catalytic protein tyrosine phosphatase domain. CD45 protein expression measured by FACS was slightly reduced in two of the three mutated cases (not shown).

Finally, 9% of T-PLL in our cohort carried mutations in *FOXP1*, encoding a ubiquitously expressed transcription factor and essential regulator in human CD4^+^ T cells^31,32^. All mutations, including missense and nonsense mutations, are located in the forkhead domain, which mediates monomeric DNA binding^33^.

### Copy number variations

To identify gains and losses in this T-PLL cohort, we performed genome-wide CNV analyses for 14 patients (Table 2). Besides already known CNV, we identified several previously unknown gains and losses as well as uniparental disomies (UPDs) for the 40 genes of interest (Table 2). Two of fourteen (14%) patients displayed losses of the *SAMHD1* locus at 20q11. In addition, we identified two further patients carrying UPDs involving *SAMHD1*. None of these four patients had a point mutation or small indel in *SAMHD1*. For *HERC2*, *HERC1*, *FOXP1*, and *PRDM2*, no copy number alterations were observed.

**Table 2:** Copy number variations and UPDs for 40 selected genes

**Array**		**SNP 6.0**										**CytoHD**			
**Gene**	**Chr**	**T-PLL 1**	**T-PLL 8**	**T-PLL 2**	**T-PLL 7**	**T-PLL 10**	**T-PLL 4**	**T-PLL 38**	**T-PLL 11**	**T-PLL 5**	**T-PLL 3**	**T-PLL 33**	**T-PLL 34**	**T-PLL 35**	**T-PLL 36**
ARID4B	1														
JAK1	1														
MTOR	1														
PRDM2	1														
PTPRC	1				UPD 4.7 Mb										
CEBPZ	2										Gain 46.6 Mb				
FOXP1	3														
FBXW7	4	Gain 7.6 Mb													
TET2	4														
WHSC1	4			Loss 1.8 Mb											
TNIP1	5														
BCLAF1	6					Loss 66 Mb	UPD 104 Mb				Loss 4.1 Mb				
CUX1	7					Gain 2.8 Mb	Gain 10.2 Mb				Gain 0.8 Mb				
KMT2C	7	Loss 4 Mb		Loss 1.3 Mb		UPD 8.1 Mb	Gain 5.8 Mb				Loss 3.9 Mb				Loss 14.7 Mb
KMT2E	7					Gain 0.6 Mb	Gain 1.6 Mb				Gain 3.5 Mb				Loss 0.4 Mb
VOPP1	7						Gain 9.2 Mb								
PARP10	8	Gain 2.2 Mb	Gain 5.2 Mb			Gain 4.3 Mb	Gain 34 Mb	Gain 7.1 Mb	Gain 2.2 Mb			Gain 93.1 Mb	Gain 27.2 Mb	Gain 88.3 Mb	Gain 58 Mb
NOTCH1	9														
USP20	9														
ATM	11	Loss 8.8 Mb	Loss 2.2 Mb			Loss 7.1 Mb		Loss 7.2 Mb	Loss 7.1 Mb	UPD 74.4 Mb		Loss 25.4 Mb		Loss 9 Mb	Gain mosaic 51.3 Mb
CBL	11					Loss 6.2 Mb			Loss 10.4 Mb	UPD 74.4 Mb		Loss 25.4 Mb			Gain mosaic 51.3 Mb
CCDC88B	11									UPD 74.4 Mb					
DDB2	11		Loss 2.5 Mb					Loss 1 Mb		Loss 3.5 Mb					
ETV6	12				Loss 2.8 Mb	Loss 3.3 Mb			Loss 2.3 Mb				Loss mosaic 4.5 Mb		
HIP1R	12					UPD 84.4 Mb									
KMT2D	12					UPD 84.4 Mb									
RPLP0	12					UPD 84.4 Mb									
BCL11B	14	Gain 3.4 Mb	Gain 4.2 Mb							Gain 18.5 Mb					Ggain 66.7 Mb
HERC1	15														
HERC2	15														
STAT5B	17						UPD 50.7 Mb				Gain 0.2 Mb				
SUZ12	17						UPD 50.7 Mb								
JAK3	19									UPD 19.5 Mb					
KMT2B	19														
SAMHD1	20	Loss 1.9 Mb							Loss 2 Mb						
PRMT2	21										Gain 0.1 Mb				
RUNX1	21														
EP300	22								Loss 1.5 Mb			Gain 21.9 Mb			
BCOR	X											Loss mosaic 56.8 Mb			
ZRSR2	X											Loss mosaic 56.8 Mb			

*Chr* chromosome, *UPD* uniparental disomy, *Mb* megabases

In the region containing *PTPRC*, UPDs were observed in 3/14 patients (21%). None of these patients displayed other *PTPRC* mutations. Recurrent gains were observed in *BCL11B* in 4/14 patients (29%). The role of *BCL11B*, which is a main maturation factor of T cells^34,35^, in hematologic malignancies is still discussed. In T-ALL it is reported as either a tumor suppressor or oncogene^36,37^.

### SAMHD1 protein expression is reduced in T-PLL carrying mutations or gene losses

**Fig. 4:**
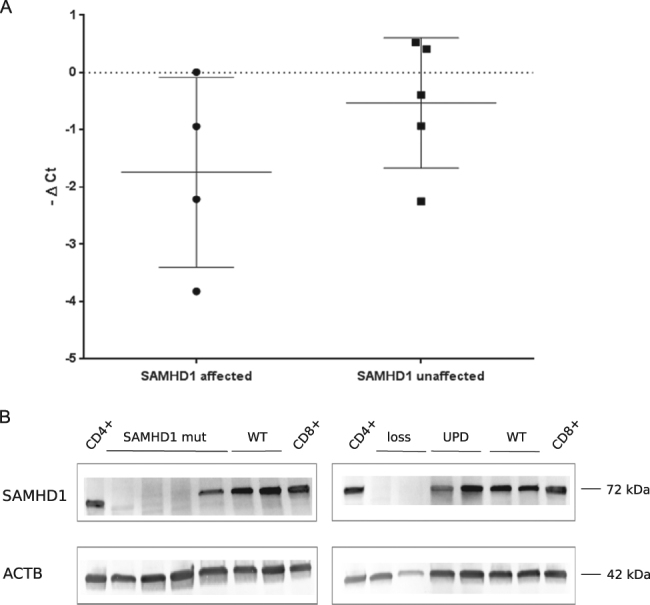
SAMHD1 mRNA and protein expression in T-PLL **a** mRNA expression levels (−ΔCT values) for SAMHD1 of T-PLL with mutated or deleted SAMHD1 (affected) compared to T-PLL with wild-type SAMHD1 (unaffected). Samples with mutated or deleted SAMHD1 have a lower SAMHD1 mRNA expression. GAPDH was used as internal reference. Bars indicate mean and S.D. **b**. Protein expression levels of 12 T-PLL samples. Samples with mutated SAMHD1 or losses show lower SAMHD1 protein expression compared to WT samples. β-actin served as loading control. *mut* mutated, *WT* wild type, *UPD* uniparental disomy, *ACTB* Actin beta

As most mutations in *SAMHD1* were destructive or caused loss of one allele, we analyzed whether these genetic lesions affected RNA and/or protein expression of SAMHD1. Quantitative real-time reverse transcription PCR showed variable mRNA levels of *SAMHD1* in nine T-PLL analyzed (Fig. 4a). Three T-PLL with *SAMHD1* point mutations or a deletion were among the five T-PLL with lowest transcript levels. However, there was no strict correlation between *SAMHD1* mRNA levels and presence of mutations. More importantly, western blot analysis of 12 T-PLL revealed that T-PLL without genetic lesions in *SAMHD1* had strong protein expression, comparable to normal peripheral blood CD4^+^ and CD8^+^ T cells, whereas three of the four mutated cases had hardly detectable levels, and one case slightly reduced levels (Fig. 4b). A similarly low expression was observed for cases with losses and even for one case with a UPD (Fig. 4b).

### T-PLL cells carrying *SAMHD1* mutations do not respond differently to cytarabine in vitro

**Fig. 5:**
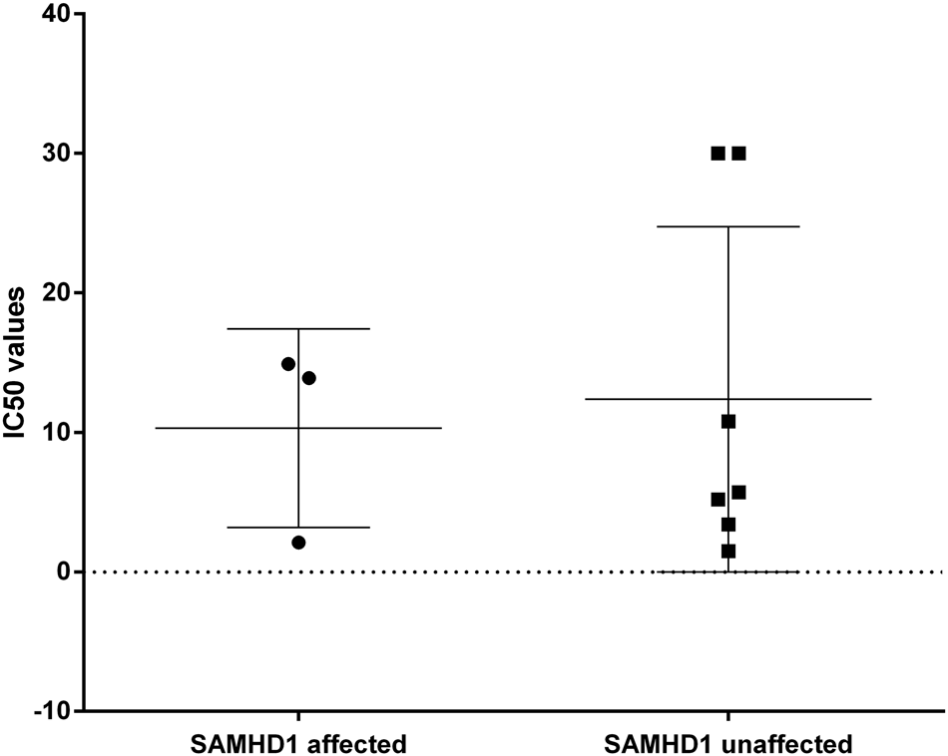
Lack of toxic effects of cytarabine on T-PLL cells IC_50_ values for cytarabine of 12 T-PLL samples separated into SAMHD1 affected (*n* = 4) and SAMHD1 unaffected (*n* = 7) cases. Bars indicate mean and S.D.

SAMHD1 reduces cytarabine (Ara-C) toxicity in acute myeloid leukemia cells^38^. Ara-CTP, which is the active triphosphate metabolite of Ara-C, is hydrolyzed by SAMHD1 as its direct substrate, which results in reduction of Ara-C levels. Hence, in cells with inactive SAMHD1, the cytotoxic activity of Ara-C is potentiated^38^. On the other hand, SAMHD1 may potentially also decrease cell sensitivity to AraC, namely due to the increase of cellular dCTP as competitor of AraCTP. The net outcome may depend on cell types. Although Ara-C is not particularly effective in T-PLL^39^, we tested drug toxicity to determine a potential effect of Ara-C in cases with *SAMHD1* mutations. We performed cell viability assays on thawed viably cryopreserved T-PLL cells after treatment with Ara-C and a number of other cytotoxic agents in different concentrations (Table 3). However, we observed no difference in the IC_50_ values between cells with low or high SAMHD1 protein expression, indicating that Ara-C is not cytotoxic for T-PLL cells, even not in those with low SAMHD1 expression (Fig. 5). The response of SAMHD1 mutant cases was not improved for any of the drugs tested. When analyzing the cases for the five most frequent mutations occurring in this cohort, we observed next to Ara-C non-responsiveness that *SAMHD1* mutant cases neither responded to fludarabine nor clofarabine. By contrast, four of the *ATM* mutated cases responded well to fludarabine and clofarabine (Table 3). Each of the four cases carried non-synonymous mutations that were predicted to be destructive for ATM protein function by at least 5/7 prediction tools. Two of the cases additionally showed deletions of the second *ATM* allele. Hence, although the functional impairment of ATM in these cases could not be experimentally validated, it seems that in these T-PLL, *ATM* mutations are not linked to resistance against fludarabine or clofarabine.

**Table 3:** Cell viability assay

	**IC50 (µM)**											
**Compound name**	**T-PLL_2**	**T-PLL_3**	**T-PLL_10**	**T-PLL_11**	**T-PLL_7**	**T-PLL_36**	**T-PLL_5**	**T-PLL_25**	**T-PLL_34**	**T-PLL_37**	**T-PLL_35**	**Mean** ^**#**^
Cytarabine	16.095	*1.875*	**3.212**	29.91	14.727	**3.897**	29.91	**7.761**	*2.034*	24.826	6.233	8.3
Fludarabine	29.91	29.91	0.468	29.91	29.91	0.76	25.828	***39.91***	0.241	29.91	0.363	6.4
Clofarabine	29.91	29.91	0.046	29.91	29.91	0.225	29.91	29.91	0.372	29.91	NA	6.2
Cyclophosphamide	29.91	29.91	29.91	29.91	29.91	29.91	29.91	29.91	29.91	29.91	29.91	29.9
Prednisone	ND	ND	ND	29.91	29.91	29.91	29.91	29.91	29.91	29.91	29.91	29.9
Doxorubicine	0.226	*2.523*	3.535	4.011	4.208	4.532	5.645	*1.935*	*2.061*	0.553	5.439	2.3
**SAMHD1 status**	Mutated	Mutated	WT	Loss	WT	Loss (UPD)	WT	WT	WT	Mutated	WT	
**ATM status**	WT	WT	Mutated/loss	Mutated	Mutated	Mutated/gain	Mutated	WT*	Mutated	WT*	Mutated/loss	
**JAK3 status**	WT	Mutated	WT	WT	WT	Mutated	Mutated/UPD	*	WT	Mutated*	WT	
**HERC2 status**	Mutated	WT	WT	WT	WT	WT	WT	*	WT	*	WT	
**FOXP1 status**	WT	WT	WT	WT	WT	WT	WT	*	Mutated	*	WT	

*NA* not available, *ND* not determined, *WT* wild type, *UPD* uniparental disomy.

^*^No copy number status available;

^**#**^calculated as geometric mean

Correlating *SAMHD1* mutations to clinical data, we observed no differences between mutated and unmutated cases in terms of age at diagnosis, genetic group, T-PLL immunophenotype, absolute white blood cell count, and absolute lymphocyte count at diagnosis, sites of involvement or response to therapy (Table 1).

### Intracellular dNTP levels of *SAMHD1*-mutated T-PLL are elevated compared to *SAMHD1* wild-type and healthy blood donor-derived CD3^+^ samples

**Fig. 6:**
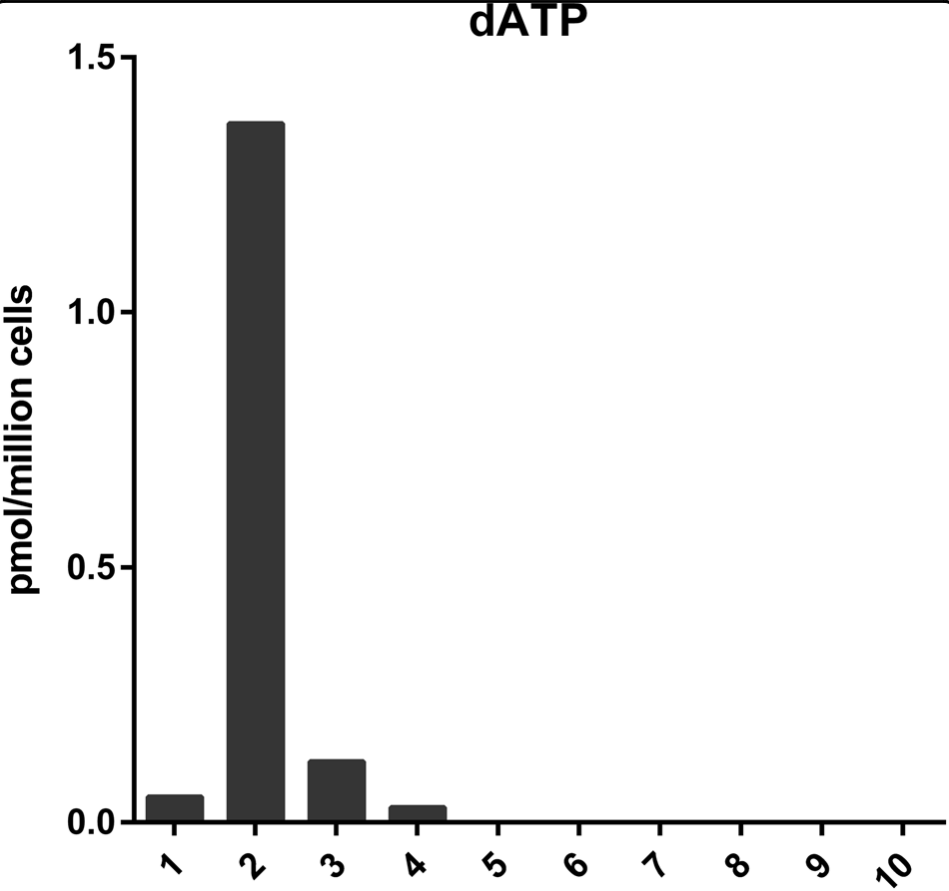
dATP levels of T-PLL and healthy donor samples Display of dATP levels from dNTP measurement from 2 × 10^6^ cells. Samples 1–4: T-PLL with *SAMHD1* mutations. Samples 5–7: T-PLL with wild-type *SAMHD1*. Samples 8–10: healthy donor-derived CD3^+^ T cells. In samples 5–10 the dNTP levels were not detectable

Intracellular dNTP levels measured from 2 × 10^6^ cells of four *SAMHD1*-mutated samples revealed detectable levels, whereas no dNTPs were detected in three T-PLL *SAMHD1* wild-type samples and three healthy donor-derived CD3^+^ samples, using the same cell numbers for analysis (Fig. 6). The dNTP levels did not correlate with the size of the *SAMHD1* mutant clone. The contingency analysis revealed a significant difference between *SAMHD1*-mutated and unmutated samples (*p* = 0.0048, two-tailed Fisher’s exact test).

## Discussion

T-PLL is characterized by inversions or translocations involving the *TCL1* gene or the *MTCP1* gene, along with one of the TCR loci^3–6^. Further genetic lesions involved in T-PLL pathogenesis involve *ATM*^7–9^, members of the JAK/STAT signaling pathway, and epigenetic regulators^7,11,12,40^.

We focused here on 40 candidate genes selected from an exploratory RNA-Seq analysis. Formally, we cannot exclude that a few alterations identified in the RNA or DNA analyses are not somatic mutations but represent germline variants, because for most cases non-tumor RNA or DNA was not available. However, our stringent filtering against known polymorphisms, the occurrence of distinct non-synonymous mutations for recurrently mutated genes, and the direct experimental verification for the somatic origin of three of four tested *SAMHD1* mutations together argue that the vast majority of events described here are indeed somatic mutations. Overall, our results confirm previously reported data from our^12^ and other groups^7^ and furthermore reveal novel recurrently altered genes in T-PLL (Fig. 1).

Most recurrently altered genes encode proteins with functions in DNA damage/repair. This category included *HERC2* and *HERC1*, the latter of which has been previously reported as recurrently mutated in up to 13% of patients with T-ALL^22^. We observed nonsense and missense mutations in the ubiquitin-interacting motif of *PARP10*. As PARP10 deficiency leads to severe DNA repair defects^41^, these mutations may disturb DNA repair in T-PLL. Ten of fourteen patients (71%) displayed gains involving the *PARP10* gene. Such gains are in a considerable number of cases occurring due to formation of an isochromosome i(8q). While the point mutations observed in *PARP10* likely have functional consequences, it remains unclear whether these gains translate into higher protein levels and hence functional alterations.

A further gene in the category of DNA damage/repair is *SAMHD1*, which is mutated in 6/33 (18%) of cases and, moreover, is affected by deletions in two further cases. *SAMHD1* encodes a dGTP-activated triphosphohydrolase, which regulates the cellular dNTP pool^42^. Inactivating mutations in *SAMHD1* can promote tumor cell survival as they lead to increased dNTP levels^42^. *SAMHD1* is recurrently mutated in CLL, and the protein is supposed to act as a tumor suppressor^43^. Most mutations we observed in *SAMHD1* are frameshift or nonsense mutations. In several cases, the high VAF of mutations indicates biallelic destruction of the gene. SAMHD1 is highly expressed in monocytes, macrophages, dendritic cells, and resting T cells^44,45^. In T-PLL, we observed variable SAMHD1 protein expression levels, but in general protein expression of wild-type SAMHD1 was comparable to resting normal CD4^+^ and CD8^+^ T cells (Fig. 4b). By contrast, T-PLL cases carrying *SAMHD1* mutations or losses showed hardly any SAMHD1 protein, further validating the destructive nature of the mutations (Fig. 4b). We also observed downregulation of SAMHD1 protein in four T-PLL cases not carrying mutations or losses in the coding region of this gene (data not shown). In these cases, epigenetic silencing might occur, although at the group level we did not observe hypermethylation of the *SAMHD1* promotor by comparing over 50 T-PLL to normal T-cell subsets (data not shown; to be published elsewhere). Thus, the situation in T-PLL is different from that of CD4 T cells of patients with Sézary syndrome in which the *SAMHD1* promotor is hypermethylated^46,47^. We did not observe a stringent correlation between SAMHD1 protein and mRNA expression (Fig. 4a), which hints toward a regulation of gene expression on different levels. Notably, in the present cohort *SAMHD1* mutations are significantly negatively associated with *ATM* mutations (Fisher’s exact test, *p* = 0.02). Hence, SAMHD1 inactivation may be an alternative lesion to loss of ATM function to impede proper DNA repair in T-PLL cells. The observation that *SAMHD1* mutant cases did not show differences to wild-type cases regarding clinical data might be explained by the relatively low number of cases analyzed, but even more by the aggressive nature of T-PLL and short survival of patients with this disease, so that moderate alterations of tumor cell physiology do not translate into a significant change of clinical outcome.

SAMHD1 regulates dNTP homeostasis by decreasing intracellular dNTP levels. In T-PLL, we observed elevated dNTP levels in *SAMHD1-*mutated cases compared to *SAMHD1* wild-type cases and CD3^+^ T cells from healthy donors, confirming that mutant *SAMHD1* is not able to regulate dNTP levels in T-PLL (Fig. 6). Nucleoside analogs, which are used as therapeutic agents against leukemias, are incorporated into the DNA by a competitive mechanism. It was supposed that in leukemias *SAMHD1* mutations causing loss of the proteins’ tumor-suppressive function could lead to enhanced drug efficacy^48,49^. However, for patients with acute myeloid leukemia it was observed that Ara-CTP, the active metabolite of cytarabine, is a direct substrate of SAMHD1. Hence, intact SAMHD1 reduces Ara-C levels, whereas impaired SAMHD1 protein leads to more effective drug responses^38^.

Although Ara-C is not generally effective in T-PLL, we analyzed whether T-PLL with *SAMHD1* mutations or deletions show an increased sensitivity toward cytarabine. However, this was not observed (Fig. 5). Testing of further nucleoside analogs including Fludarabine and other drugs showing cytotoxic effects in T-PLL also revealed no increased sensitivity in *SAMHD1* mutant cases toward any of the drugs (Table 3). Therefore, we conclude that the disturbance of the dNTP pool caused by *SAMHD1* mutations in T-PLL leads to a benefit for the tumor cells, since increased intracellular dNTPs promote cell cycle progression, proliferation, and survival of the cells. Regarding the non-responsiveness to drugs, in T-PLL further regulatory mechanisms are likely playing a major role in this context, reflecting the overall drug resistance of these tumor cells.

In the present cohort, *PRDM2* is the most frequently mutated gene among those involved in epigenetic regulation. *PRDM2* is frequently inactivated by mutations in colorectal cancer cell lines and in relapsed bladder cancer^25,26^. The amino-acid changing mutations we observed, occurring in the coiled-coil domain of the protein, lead most likely to an impaired function, potentially resulting in tumor cell advantage. We also validate the occurrence of mutations in other epigenetic regulators, namely members of the KMT2 family, BCOR, and TET2.

*PTPRC*, encoding CD45, is recurrently mutated in T-ALL, leading to downregulation of the protein^30^. Two of three mutations we observed were located in the catalytic protein tyrosine phosphatase domain, suggesting functional consequences. CD45 negatively regulates JAK/STAT signaling by dephosphorylation of all four JAKs^50^. Therefore, mutations in *PTPRC* can enhance the already constitutively activated JAK/STAT signaling in T-PLL^12^.

FOXP1 is a ubiquitously expressed transcription factor and essential regulator in human CD4^+^ T cells^32^. Main mechanisms leading to FOXP1 protein overexpression in cancers are translocations, amplifications, or the repression of miRNAs normally downregulating FOXP1 translation^51^. All three replacement mutations observed in our T-PLL cohort are located in the DNA-binding forkhead domain, with unclear consequences. Notably, however, in other T-cell lymphoproliferative disorders FOXP1 expression is constitutionally repressed^32^. Clearly, the role of FOXP1 alterations in T-PLL requires further investigations.

Novel recurrently mutated genes identified by WES included the ryanodine receptor 3 gene *RYR3*, *PARN*, coding for a poly(A)-specific ribonuclease and *PCLO*, encoding the piccolo presynaptic cytomatrix protein. RYR3 releases calcium from the endoplasmic reticulum. Intracellular calcium homeostasis plays an important role in cell metabolism, therefore a disturbed calcium metabolism caused by mutations in *RYR3* or overexpression of RYR3 may be oncogenic, as reported for several cancers^52^. As the point mutations we observed are not located in already described domains, their impact is unclear. However, the observation of a fourfold higher expression of the *RYR3* gene in T-PLL compared to normal CD3^+^ T cells (*p* = 0.026) is in line with altered *RYR3* expression in several cancer types^52^. *PARN* was already described as a potential tumor-suppressor gene^53^. Two missense mutations are located directly in front and behind a coiled-coil region of PARN, which may alter its function. *PCLO* is recurrently mutated or targeted by copy number gains in diffuse large B-cell lymphomas^19,20^. We identified one missense and one frameshift mutation, and two copy number gains in two further patients. These seemingly contradictory findings (inactivating frameshift mutation vs. increased gene dosage by copy gain) warrant further investigations.

In conclusion, we identified novel recurrently mutated genes in T-PLL, including *PTPRC*, regulating the JAK/STAT pathway, epigenetic regulators like *PRDM2* and *HERC1/2*, and genes involved in DNA damage response and DNA repair like *SAMHD1*, which has most likely a tumor-suppressor function in T-PLL.

## Electronic supplementary material

Supplementary Methods

Supplementary Table S1

Supplementary Table S2
