# Supplementary material for: SAMHD1 is recurrently mutated in T-cell prolymphocytic leukemia
Source: Blood Cancer J. 2018 Jan 19;8(1):11. doi: 10.1038/s41408-017-0036-5 (PMC5802577; doi:10.1038/s41408-017-0036-5)
Supplement: Supplementary file 1 — Supplementary Methods [file 41408_2017_36_MOESM1_ESM.docx]

**Supplement to**

***SAMHD1* is recurrently mutated in T-cell prolymphocytic leukemia**

Patricia Johansson^1,2^, Ludger Klein-Hitpass^2^, Axel Choidas^3^, Peter Habenberger^3^, Bijan Mahboubi ^4^, Baek Kim^4^, Anke Bergmann^5^, René Scholtysik^2^, Martina Brauser^2^, Anna Lollies^2^, Reiner Siebert^5,6^, Thorsten Zenz^7,8^, Ulrich Dührsen^1^, Ralf Küppers^2,8^ and Jan Dürig^1,8^

^1^Department of Haematology, ^2^Institute of Cell Biology (Cancer Research), University Hospital Essen, University of Duisburg-Essen, Essen, Germany, ^3^Lead Discovery Center GmbH, Dortmund, Germany, ^4^Center for Drug Discovery, Department of Pediatrics, Emory Center for AIDS Research, Emory University, Children's Healthcare of Atlanta, Atlanta; ^5^Institute for Human Genetics, Christian-Albrechts-University Kiel & University Hospital Schleswig Holstein, Kiel, Germany, ^6^Institute of Human Genetics, University of Ulm and University Hospital of Ulm, Ulm, Germany, ^7^Department of Molecular Therapy in Haematology and Oncology, National Center for Tumor Diseases and German Cancer Research Center and Dept. of Medicine V, University Hospital Heidelberg, Heidelberg, Germany, ^8^German Cancer Consortium (DKTK), Heidelberg, Germany

**Supplementary Methods**

**RNA and DNA isolation**

RNA was extracted with TRIzol (Life Technologies, Darmstadt, Germany) and purified with the RNeasy Mini Kit® (Qiagen, Hilden, Germany). DNA was extracted using the QIAamp DNA blood midi kit (Qiagen). RNA and DNA concentrations were measured with a Qubit Fluorometer 2.0 (Life Technologies). Quality assessment was performed with the Agilent Bioanalyzer 2100 (Agilent, Santa Clara, USA). Preparations were carried out according to the manufacturer’s guidelines. Between 1.5 - 17 µg of DNA, and 2 - 20 µg of RNA were isolated per sample.

**Tumor cell enrichment**

We performed density gradient centrifugation of heparinized peripheral blood samples (Pancoll human, PAN-biotech, Aidenbach, Germany). Tumor cells were enriched from peripheral blood mononuclear cells (PBMC) by fluorescence activated cell sorting (FACS Aria III, BD Biosciences; Heidelberg, Germany) after staining with antibodies against CD3 and CD4 (BD Biosciences). For cases with high tumor cell content (≥90%), tumor cells were enriched by magnetic cell separation with an anti-CD3 antibody (MACS, Miltenyi Biotech). Tumor cell content was assessed flow-cytometrically, showing 95% for one case, and for the others 98% to 100%.

**Transcriptome sequencing**

The TruSeq RNA Library Preparation Kit v2 (Illumina Inc., San Diego, CA, USA) was used to prepare sample libraries. TopHat2 was used for alignment,^1^ Strand Avadis NGS software^2^ was used for variant calling.

**Targeted capture sequencing**

The libraries were prepared with NEBNext Ultra DNA library prep kit (NEB, Frankfurt/ Main, Germany), followed by capturing with capture probes for all coding exons of the selected genes (SeqCap choice EZ, Roche NimbleGen, Madison, WI, USA). Sequencing was carried out on an Illumina HiSeq 2500 system with 2x101 bp paired-end reads. For de-multiplexing and adapter trimming CASAVA v1.7 (Illumina) and Trimmomatic,^3^ respectively, were used. The alignment was performed with Bowtie2 (ref. ^4^) against GRCh37 (hg19). Strand Avadis NGS software was used for variant calling.

**Whole exome sequencing**

Libraries were prepared from isolated tumor cells with NEBNext Ultra DNA library prep kit (NEB). Exome sequencing was carried out with NimbleGen SeqCap EZ enrichment kit v3 (Roche NimbleGen) on the HiSeq 2500 system with 2x101 bp paired-end reads (Illumina). For de-multiplexing and adapter trimming CASAVA v1.7 (Illumina) and Trimmomatic^3^ were used. Read duplicates were removed with SAMtools rmdup.^5^ The alignment was performed with BWA^6^ against GRCh37 (hg19). Strand Avadis NGS software was used for variant calling.

**Prediction tools used for results of capture and whole exome sequencing**

To identify the potential impact of mutations we only considered mutations as pathogenic, if five of seven predicition tools yielded concordant results. Predictions tools used were SIFT, PolyPhen2 HDIV, PolyPhen2 HVAR, LRT, MutationTaster, PhyloP and GERP++.^7-12^

**Copy number analysis**

Copy number variation (CNV) analysis was carried out on Affymetrix SNP 6.0 microarrays (n=10), and CytoScan HD arrays (n=4) (Affymetrix, Santa Clara, CA, USA). CNVs were analyzed with the Genotyping console (SNP 6.0) or ChAS (CytoScan HD, Affymetrix). Uniparental disomies (UPDs) were considered, if the size of a region with a copy number neutral loss of heterozygosity was larger than 3 Mb. The observed range was between 4.7 and 104 Mb (mean 39.7 Mb). The average number of probes covered within these UPDs was 14,617 (range 1,293-31,781). Data are available under accession number GSE100844.

**Western blotting**

The following primary antibodies were used: mouse monoclonal anti-SAMHD1 (1F9, dilution 1:2000; Abcam, Cambridge, UK) and monoclonal anti-β-actin-HRP (AC-15, dilution 1:500 000; Sigma-Aldrich, St. Louis, MO, USA). As secondary antibody against the anti-SAMHD1 antibody a goat-anti-mouse-HRP (115-036-062, dilution 1:4000; Jackson Immunoresearch, West Grove, PA, USA) was used.

**Quantitative reverse transcription PCR**

Each diluted reverse transcription reaction (1:20) was combined with TaqMan® Universal PCR Master Mix, no AmpErase® UNG (Thermo Fisher) and TaqMan probes for SAMHD1 (Hs00210019_m1) and GAPDH (Hs02758991) (Thermo Fisher). Real-time PCR was performed using AriaMX (Agilent genomics, Santa Clara, CA, USA).

**Cell viability assay**

Compounds/DMSO were prediluted at different concentrations in 25 µl cell culture medium in white 384 well-plates (Greiner Bio-One, Frickenhausen, Germany) on day 1. After incubation for 3 h at 37°C/5% CO_2_, 10 µl of cell suspension is added at cell numbers that assure assay linearity and optimal signal intensity. Cells were further incubated in humidified chambers for 48 h at 37°C and 5% CO_2_. Cells treated with the compound vehicle DMSO were used as positive controls and cells treated with 10 µM Staurosporine serve as negative controls.

At day 3 the CellTiter Glo reagent was prepared according to the instructions of the kit (Promega): Reagent was mixed 1:1 with cell culture medium. Thereon, mixture and assay plates were equilibrated at room temperature for 20 min. Equal volumes of the reagent-medium-mixture was added to the volume of culture medium present in each well. The plates were mixed at ~300 rpm for 2 minutes on an orbital shaker. The microplates were then incubated at room temperature for 10 minutes for stabilization of the luminescent signal. Following incubation the luminescence was recorded on a Victor microplate reader (Perkin Elmer, Waltham, MA, USA) using a 200 ms integration time. The data were then analyzed with Excel using the XLFIT Plugin (dose response Fit 205) for IC_50_-determination. As quality control the Z´-factor was calculated from 16 positive and negative control values. Only assay results showing a Z´-factor ≥ 0.5 were used for further analysis.

**References**

1. Kim D, Pertea G, Trapnell C, Pimentel H, Kelley R, Salzberg SL. TopHat2: accurate alignment of transcriptomes in the presence of insertions, deletions and gene fusions. *Genome Biology*. 2013; **14**: R36.

2. Strand NGS. San Francisco: Strand Life Sciences. Strand Genomics Inc.; 2012.

3. Bolger AM, Lohse M, Usadel B. Trimmomatic: a flexible trimmer for Illumina sequence data. *Bioinformatics*. 2014; **30**: 2114-2120.

4. Langmead B, Salzberg SL. Fast gapped-read alignment with Bowtie 2. *Nat Methods*. 2012; **9**: 357-359.

5. Li H, Durbin R. Fast and accurate short read alignment with Burrows-Wheeler transform. *Bioinformatics*. 2009; **25**: 1754-1760.

6. Li H, Handsaker B, Wysoker A, et al. The Sequence Alignment/Map format and SAMtools. *Bioinformatics*. 2009; **25**: 2078-2079.

7. Adzhubei IA, Schmidt S, Peshkin L, et al. A method and server for predicting damaging missense mutations. *Nat Methods*. 2010; **7**: 248-249.

8. Chun S, Fay JC. Identification of deleterious mutations within three human genomes. *Genome Res*. 2009; **19**: 1553-1561.

9. Davydov EV, Goode DL, Sirota M, Cooper GM, Sidow A, Batzoglou S. Identifying a high fraction of the human genome to be under selective constraint using GERP++. *PLoS Comput Biol*. 2010; **6**: e1001025.

10. Kumar P, Henikoff S, Ng PC. Predicting the effects of coding non-synonymous variants on protein function using the SIFT algorithm. *Nat Protoc*. 2009; **4**: 1073-1081.

11. Pollard KS, Hubisz MJ, Rosenbloom KR, Siepel A. Detection of nonneutral substitution rates on mammalian phylogenies. *Genome Res*. 2010; **20**: 110-121.

12. Schwarz JM, Rodelsperger C, Schuelke M, Seelow D. MutationTaster evaluates disease-causing potential of sequence alterations. *Nat Methods*. 2010; **7**: 575-576.
