# Supplementary material for: SAMHD1 is recurrently mutated in T-cell prolymphocytic leukemia
Source: Blood Cancer J. 2018 Jan 19;8(1):11. doi: 10.1038/s41408-017-0036-5 (PMC5802577; doi:10.1038/s41408-017-0036-5)
Supplement: Supplementary file 2 — Supplementary Table S1 [file 41408_2017_36_MOESM2_ESM.docx]

**Table S1. Assignment of patient data to experiments**

| **Patient**  **ID** | **Material** | **Sex** | **Years**  **at diagnosis** | **Genetic**  **Group** | **Karyotype** | **FISH** | | | **Analysis type** | | | |
| --- | --- | --- | --- | --- | --- | --- | --- | --- | --- | --- | --- | --- |
|  |  |  |  |  |  | ***TCR*** | ***TCL1*** | ***MTCP1*** | ***RNA-seq*** | ***Capture*** | ***Exome-Seq*** | ***SNP array*** |
| **1** | PB | F | 74 | inv(14)/  t(14;14) | n.a. | break | break | n.a. | x | x |  | x |
| **2** | PB | F | 64 | t(X;14) | 45,X,t(X;14)(q28;q11),der(1)t(1;9)(q21;q12),der(7)t(1;7)(q21;q32),−9[20] | break | normal | break | x | x |  | x |
| **3** | PB | M | 62 | inv(14)/  t(14;14) | 45,XY,add(5)(q31),der(6)t(Y;6)(q11;q12),dup(7)(q35q31),add(9)(p23),der(11)t(11;17)(q25;q21), der(13)t(6;13)(p21;p13)del(13)(q13q21),add(14)(p13),del(14)(q11q32),dup(17)(q11q23),−17[23]/ 46,XY[2] | break | break | n.a. | x | x |  | x |
| **4** | PB | M | 69 | inv(14) | 49,XY,+7,der(8)t(6;8)(p21;p21),+der(8)t(8;8)(p21;q21),+del(8)(p11),inv(14)(q11q32)[13] | break | break | n.a. | x | x |  | x |
| **5** | PB | M | 75 | inv(14) | 43,X,t(Y;14)(q10;q10),t(1;5)(p36;p14),der(7)t(7;11)(p22;q12),r(8)(p23q24),−11,inv(14)(q11q32),−17,der(19)t(14;19)(q32.1;p13.3)x2[14] | break | break | n.a. | x | x |  | x |
| **6** | PB | F | 41 | t(X;?) | 43~45,X,-X[6],dic(6;11)(q11;q13)[5],add(8)(p23)[2][cp6]/46,XX[7] | normal | normal | break | x | x |  |  |
| **7** | PB | F | 51 | t(14;14) | 42~43,XX,add(5)(p14),der(9)t(9;11)(p21;p12~14)add(9)(q12),del(11)(p12~14),der(12)t(12;22) (p11;q13),t(14;14)(q11;q32),-18,der(18)t(5;18)(p14;q22),−20,−22[cp10] | break | break | n.a. | x | x |  | x |
| **8** | PB | M | n.a. | t(X;14) | 46,t(X;14)(q27;q11),Y,der(3)add(3)(p24)add(3)(q12),add(3)(q21),dup(8)(q21q24),-10,-11,der(22)t(14;?;22)(q21;?;p13),+2mar[12] | break | normal | break | x | x |  | x |
| **10** | PB | M | 82 | inv(14) | 44,XY,i(6)(p10),i(7)(q10),der(7)t(7;7)(p11;q35),i(8)(q10),−10,add(10)(p11),del(11)(q14q24),del(12) (p12),inv(14)(q11q32),−17[20] | break | break | n.a. | x | x |  | x |
| **11** | PB | F | 62 | inv(14) | 42~44,XX,del(6)(q12),+7,der(7;11)(p10;q10),ider(8)(q10)add(8)(q24),der(11;20)(p10;q10),add(12) (p12),−13,−14,inv(14)(q11q32),add(16)(p13),−21,−22,+mar[cp8]/46,XX[2] | break | break | n.a. | x | x |  | x |
| **12** | PB | M | 57 | inv(14) | 43~46,X,−Y,der(8)t(8;8)(p21;q21),add(10)(p1?2),−13,inv(14)(q11q32),add(18)(q23),−22,+1~2mar [cp14]/46,XY[5] | n.e. | n.e. | n.e. |  | x |  |  |
| **13** | PB | F | 53 | inv(14) | 44,XX,dic(1;11)(q10;q13~14),der(4)t(1;4)(p31;q32),der(6)del(6)(q12q16)del(6)(q24q25),+9,add(9)(p13),inv(14)(q11q32),−18,der(18;21)(q10;q10),+19,−22[17]/46,XX[3] | break | break | n.a. |  | x |  |  |
| **15** | PB | F | 78 | t(X;14) | 45,X,t(X;14)(q28;q11),der(3)t(3;8)(q27;q24),der(6)i(6)(p10)t(6;22)(p21;q12),der(10)t(10;11) (p12;p11),?dic r(16;22)(p13.3q23;p13q12)[20] | break | normal | break |  | x |  |  |
| **16** | BM | M | 47 | inv(14) | 42~44,X,−Y,del(2)(q24q32),+8,add(11)(q21),del(12)(p12),+add(12)(p12),add(13)(p13),−13,−14, inv(14)(q11q32.1), +i(16)(p10),−18,−20,−21,+mar[cp12] | break | break | n.a. |  | x |  |  |
| **17** | PB | M | 48 | inv(14) | 45,X,−Y,i(6)(p10),del(7)(p15),der(8)t(8;8)(p21;q21),−10,der(11)del(11)(q22q23)dup(11)(q22q24), dic(12;15)(p12;p13),add(13)(p13),inv(14)(q11q32),add(15)(q24),der(21)t(10;21)(q11;p13),add(22) (p13),+der(?)t(?;8)(?;q21)[cp21] | break | break | n.a. |  | x |  |  |
| **18** | PB | M | n.a. | i8(q10) | 45,X,add(Y)(p11),add(5)(q34),i(8)(q10),del(11)(q23),dic(13;22)(p13;p13) | n.a. | n.a. | n.a. |  | x |  |  |
| **19** | PB | F | 54 | inv(14) | 45,XX,der(5)t(5;11)(p14;q13),i(8)(q10),−11,t(12;14)(p12;q12~13),−14,inv(14)(q11q32),add(17)(p12),add(18)(p11),+mar[cp 15] | break | break | n.a. |  | x |  |  |
| **20** | BM | F | 55 | inv(14) | 44,XX,t(1;2)(q32;q35),der(5)t(5;14)(q13;q24)inv(14)(q11q32),i(8)(q10),der(14)inv(14)(q11q32) t(5;14)(q13;q24),−20,−22[15]/46,XX[6] | break | break | n.a. |  | x |  |  |
| **21** | PB | M | n.a | inv(14) | nuc ish 8 (CEP 8 x 2, MYC x 3~4, MYC prox x 3~4, MYC dist x 3~4), 11 (CEP11 x 2, ATM x 2, FDX x 2), 13q14 (RB x 1), 14q11 (TCR A/D prox x 2, TCR A/D dist x 2), 14q32 (IGH x 2, TCL1 prox x 2, TCL1 dist x 2), 21q (LSI 21 x 2) (MYC prox sep MYC dist x 0)(MYC con IGH 0) (TCR A/D prox sep TCR A/D dist x 1) (TCL1 prox sep TCL1 dist x 1) | break | break | n.a. |  | x |  |  |
| **22** | PB | F | 69 | inv(14) | 43,XX,−9,i(8)(q10),inv(14)(q11.2q32.1),der(14;15)(q10;q10),t(15;16)(q15;q24),−20[9]/46,XX,der(6) t(3;6)(p14;q21),inv(14)(q11.2q32.1),der(16)inv(16)(p13q11)inv(16)(q11q24),der(21)t(21;21) (p11;q21)[5] | break | break | n.a. |  | x |  |  |
| **24** | PB | M | n.a | t(14;14) | 46,XY,der(8)t(8;8)(p21;q21),add(12)(p13),+14,der(14)(14pter->14q10::14q22->14q11::14q32->14qter)x2,-18,add(19)(p13),der(22)t(?12;22)(q14;q11)[29]/46,XY[2] | break | break | n.a. |  | x |  |  |
| **25** | PB | F | 76 | inv(14) | 46,XX,der(11)t(8;11)(q11;q11),inv(14)(q11;q32)/ 46,XX,der(11)t(8;11)(q11;q11)der(12)t(12;16)(p11;p11) inv(14)(q11;q32)del(15)(q21;q25);der(16)t(12;16)(p13;p11) | break | break | n.a. |  | x |  |  |
| **26** | PB | M | n.a. | inv(14) | 44,X,-Y,t(4;9)(q22~24;q34),add(6)(q12),+8,der(8)t(8;8)(p21;q21)x2,-11,inv(14)(q11q32),del(17)(q24),add(19)(p13),der(21;22)(q10;q10)[7]/46,XY[11] | break | break | n.a. |  | x |  |  |
| **28** | PB | F | n.a. | inv(14) | 44, X, add(X)(q25), del(3)(p11), der(4)t(3;4)(p12;p15), der(8)?t(X;8)(q25;p22), der(8)t(8;8)(p22;q23), -11, -13, inv(14)(q11q32), r(17)(p11q24) [cp 9] / 44, XX, inv(1)(p12q25~31), -11, -13, inv(14)(q11q32), add(16)(q11), r(17)(p11q24) [13] |  | break | n.a. |  | x |  |  |
| **29** | BM | M | n.a. | inv(14) | 40,X,-Y,add(2)(p12),der(6)t(6;8)(q25;q21),der(7)t(2;7)(p13;q31),der(9)t(?8;9)(q21;p21),der(10;22),dic(11;17)(p11;p12),der(12)t(?8;12)(q22;p11),-13,der(13;18)(q10;q10),-14,inv(14)(q11q32),i(15)(q10),der(15;22)(q10;q10)[15]/40,X,-Y,add(2)(p12),r(3)(?),der(6)t(6;8)(q25;q21),der(7)t(2;7)(p13;q31),i(8)(q10),der(10;22),dic(11;17)(p11;p12),der(12)t(?8;12)(q22;p11),der(13;14)(q10;q10),der(13;16)(pq10;q10),inv(14)(q11q32),i(15)(q10),der(15;22)(q10;q10)[10] | break | break | n.a. |  | x |  | x |
| **30** | BM | F | n.a. | t(X;14) | 44~45,X,t(X;14)(q26;q11),+4,der(8)t(8;8)(p21~22;q21),-10,del(11)(q14q24),-13,der(15;22)(q10;q10),-18,der(21)t(14;21)(q11;p13),idic(21)(p13),+1~2mar[cp14]/46,XX[9] | break |  | break |  | x |  |  |
| **31** | PB | F | n.a. | inv(14) | 45,X,-X,+10,der(10;18)(q10;q10),inv(14)(q11q32)[1] /46,idem, der(9)t(9;14)(p21;q31)inv(14)(q11;q32),der(14)inv(14)(q11;q32)t(9;14)(p21;q31),+16[12] | 30% | break | n.a. |  | x |  |  |
| **33** | PB | F | 64 |  | 46, XX (one metaphase only) | break | n.a. | n.a. |  |  | x | x |
| **34** | PB | M | 71 |  | n.a. | break | n.a. | n.a. |  |  | x | x |
| **35** | PB | M | 76 | inv(14) | 46,XY,add(8)(p23), i(8)(q10)[2], 46, XY, +8, add(8)(p23), i(8)(q10),-11,inv(14)(q11q23)[3],47XY,+8 add(8)(p23),i(8)(q10)x2, -11,inv(14)(q11q23)[3],46,XY [6] | break | n.a. | n.a. |  |  | x | x |
| **36** | PB | M | 70 | t(X;14) | 42-43,der(X),t(X;14)(q28;q11),-Y,add(4)(p16),7,add(7)(q36),der(1;14)(q10;q10), der(10;14)(q10;q10),der(11)(t11;?;14)(p15;?;q11),+1~3mar,inc[cp8]/46,XY [7] | break | n.a. | break |  |  | x | x |
| **37** | PB | M | 74 | inv(14) | 46,XY,inv(14)(q11q32)[5],45,idem,der(1)t(1;8)(q31;q12),-8,der(12)t(1;12)(q31;p13)[14],46,XY[1] | break | break | n.a. |  |  | x |  |
| **38** | PB | F | 74 | inv(14) | 46,XX,−7,+8,der(8)t(8;8)(p21;q23),dic(11;?)(p11;?)t(?;7)(?;q21),inv(14)(q11q32),der(19)t(13;19) (q31;p13)[8] | break | break | n.a. |  | x |  |  |

PB, peripheral blood; BM, bone marrow; n.a., not available
